# Linc00665 Can Predict the Response to Cisplatin-Paclitaxel Neoadjuvant Chemotherapy for Breast Cancer Patients

**DOI:** 10.3389/fonc.2021.604319

**Published:** 2021-03-02

**Authors:** Huijuan Dai, Xiaonan Sheng, Rui Sha, Jing Peng, Fan Yang, Liheng Zhou, Yanping Lin, Yaqian Xu, Shan Zhang, Wenjin Yin, Jinsong Lu

**Affiliations:** Department of Breast Surgery, Renji Hospital, School of Medicine, Shanghai Jiaotong University, Shanghai, China

**Keywords:** breast cancer, long non-coding RNA, neoadjuvant chemotherapy, predictive biomarker, pathological complete response

## Abstract

**Objective:**

Linc00665 is a novel long non-coding RNA that can promote the progression of breast cancer, but its value in predicting the efficacy of neoadjuvant chemotherapy (NAC) for breast cancer has not been reported. We aim to analyze the correlation between Linc00665 expression and pathological complete response (pCR) in breast cancer patients.

**Materials and Methods:**

The present study examined the predictive role of Linc00665 expression in pCR after NAC using both univariate and multivariate logistic regression analyses. Receiver operating characteristic (ROC) curve and area under curve (AUC) were utilized to evaluate the performance of Linc00665 in predicting pCR. The Kyoto Encyclopedia of Gene and Genome (KEGG) analysis and Gene Set Enrichment Analysis (GSEA) were also conducted to determine the biological processes where Linc00665 may participate in.

**Results:**

The present study study totally enrolled 102 breast cancer patients. The univariate analysis showed that Linc00665 level, human epidermal growth factor receptor 2 (HER2) status and hormone receptor (HR) status were correlated with pCR. The multivariate analysis showed that Linc00665 expression was an independent predictor of pCR (OR = 0.351, 95% CI: 0.125–0.936, P = 0.040), especially in patients with HR-positive/HER2-negative subtype (OR = 0.272, 95% CI: 0.104–0.664, P = 0.005). The KEGG analysis indicated that Linc00665 may be involved in drug metabolism. The GSEA analysis revealed that Linc00665 is correlated to DNA damage repair.

**Conclusion:**

Linc00665 may be a potential novel predictive biomarker for breast cancer in NAC, especially for HR-positive/HER2-negative patients.

## Introduction

Neoadjuvant chemotherapy (NAC) has been widely used for locally advanced breast cancer and inflammatory breast cancer. This is employed for patients to decrease tumor size, converting the inoperable breast cancer into an operable one (1) or making patients qualified for breast-conserving surgery (2). After NAC in breast cancer patients, pathological complete response (pCR) is a reliable indicator for not only the complete clearance of breast cancer cells (3) but also the response of primary tumors to the selected drug regimen (4).This is also closely related to the disease-free survival and overall surviva l (5). However, previous data has revealed that breast cancer patients not reaching pCR account for more than 50% (6), and the reasons why the responses to NAC vary by patient have not been explored clearly. Therefore, there is an urgent need to explore novel endogenous molecular biomarkers to predict and improve the rate of pCR after NAC.

Long non-coding RNA (LncRNAs) are more than 200 nucleotides in length, and usually do not have the ability to encode protein, and their quantity exceed the number of protein-coding genes (1). It has been reported that lncRNAs participate in a number of biological processes, such as tumorigenesis and the drug resistance of breast cancer (7). Linc00665 is located at human chromosome 19q13.12. A series of researches have revealed that Linc00665 is upregulated in a variety of cancers, and can promote tumor progression (8–11). For example, Linc00665 can promote breast cancer cell migration and invasion by acting as miRNA sponge and stimulating the process of epithelial-mesenchymal transition (EMT) (12, 13). Additionally, Linc00665 can lead to acquired resistance to gefitinib in non-small-cell lung cancer (NSCLC) patients *via* stimulating PI3K/AKT transduction pathway (14). However, few studies have been conducted to determine whether Linc00665 is associated with the resistance of treatment for breast cancer patients, especially the sensitivity to NAC in breast cancer.

Given the effect of Linc00665 in tumorigenesis, EMT progression and drug resistance, the investigators speculate that high expression of Linc00665 might make breast cancer insensitive to NAC, and not prone to achieve pCR. In order to validate this hypothesis, we conducted an exploratory retrospective analysis of breast cancer patients in our prospective clinical trials to identify the correlation between Linc00665 expression and the sensitivity to paclitaxel and cisplatin-based NAC.

## Methods

### Patients Cohort

This study is a retrospective translational study based on the prospective cohort of the SHPD002 study (ClinicalTrials.gov identifier: NCT02221999), which enrolled 102 locally advanced breast cancer patients with sufficient preoperative biopsy cancer tissues before NAC. This clinical trial obtained the approval of the Institutional Review Board of Renji Hospital, School of Medicine, Shanghai Jiaotong University. All participants signed the informed consent forms.

These participants included women who were >18 and <70 years old, and were histologically diagnosed with locally advanced invasive breast cancer. For all participants, the NAC comprised of 4 cycles of paclitaxel (80 mg/m^2^) on day 1, 8, 15, and 22 of each 28-day cycle and cisplatin (25 mg/m^2^) on days 1, 8, and 15 of each 28-day cycle, and both were given intravenously. Hormone receptor (HR) positive breast cancer patients were randomized to concurrent endocrine therapy (including letrozole for postmenopausal women and gonadotropin releasing hormone agonist for premenopausal counterparts) or not at the time of the chemotherapy. Premenopausal triple negative breast cancer patients were randomized to gonadotropin releasing hormone agonist concurrent with chemotherapy or not. Patients with human epidermal growth factor receptor 2 (HER2) positive were recommended to receive trastuzumab concurrently with the NAC. We administered trastuzumab to patients weekly at a loading dose of 4 mg/kg, followed by a maintenance dose of 2 mg/kg weekly thereafter. When NAC was finished, all patients underwent mastectomy and lymph node dissection.

### Definitions

To determine the association of Lin00665 expression level and pCR rate, we classified the cohort into Linc00665 high and low expression group using median as the cutoff. The definition for the pCR is no invasive tumor in breast and axillary lymph nodes after surgical resection. Immunohistochemical (IHC) test of estrogen receptor (ER), progesterone receptor (PR), HER2, Ki67 and fluorescence *in situ* hybridization (FISH) of HER2 for each patient were performed on the biopsy samples before NAC. HR positive was defined as ER≥1% and/or PR≥1%. HER2 positive was defined as HER2 IHC 3+ and/or FISH amplification.

### RNA Extraction and Real-Time Quantitative Reverse Transcriptase Polymerase Chain Reaction

Total RNA was extracted from fresh frozen tumor tissues (collected by the core-needle biopsy in breast cancer patients before neoadjuvant chemotherapy) utilizing TRIzol™ Reagent (Invitrogen) according to the manufacturer’s instructions. We evaluated RNA concentration and purity by using spectrophotometry (Nano-Drop Technologies, Wilmington, Delaware, USA) and then synthesized random-primed cDNA using PrimeScript RT Enzyme Mix (Takara RR036A) on a nucleic acid amplification machine (BIO RAD, T100™ Thermal Cycler), setting the operational procedure at 37°C for 30 min and 85°C for 10 s. Linc00665 levels were detected by SYBR Green qPCR (Roche) employing a LightCycler LC480 instrument (Roche) according to the following procedures: 95°C for 5 min and 42 cycles at 95°C for 10 s, and 60°C for 1 min. We triplicated each cDNA sample in 384-microwell plates. The β-actin was employed as internal control to normalize tests. The expression of Linc00665 was calculated using the 2^-△△Ct^ method. Primer sequences are listed in **Table S1**.

### Public Database and Bioinformatics Analysis

In order to identify the potential biological processes Linc00665 is involved in, we extracted the genome-wide lncRNA expression profiles for breast cancer from The Cancer Genome Atlas (TCGA) database (https://tcga-data.nci.nih.gov/). After assigning all the expressed lncRNAs in breast cancer into high expression and low expression group on the basis of median expression levels of Linc00665, we performed the Kyoto Encyclopedia of Genes and Genomes (KEGG) pathway analysis by R (version 3.6.1) and drew with the package “clusterProfiler”, “enrichplot” (15). We also analyzed enriched biological process using Gene Set Enrichment Analysis (GSEA) (16).

### Statistical Analysis

Chi-square test was used for categorical variables and *t*-test was used for continuous variables. Age was described by mean ± standard deviation (SD). Univariate and multivariate logistic regression analyses were used to analyze the correlation between the expression of Linc00665 and the efficacy of NAC. Interaction between Linc00665 and clinicopathological factors was performed in both univariate and multivariate logistic models. For clinicopathological factors, subgroup analysis was also employed. The validity of the prediction model was tested by the receiver operating characteristic (ROC) curve and the area under the curve (AUC). We used R (version 3.6.1) and SPSS (version 23.0.0.0) for statistical analysis. A Two-sided P < 0.05 was defined as statistically significant. We used R (version 3.6.1) and Adobe Illustrator (version 21.0.0.0) to draw the curve.

## Results

### Correlation Between Linc00665 Expression and Clinicopathological Features of Patients

A total of 102 patients were enrolled in this study. Among these patients, 44.1% of patients were HER2 positive, 57.8% of patients had a Ki67 expression higher than 40%, and 81.4% patients with a tumor size of over 5 cm. The pCR rate in all breast cancer patients after NAC was 34.3% (**Table 1**). There was no statistical significance between the expression level of Linc00665 and HR status, HER2 status, Ki67 level, tumor size, clinical lymph node status and menopausal status. But Linc00665 expression level was positively related to the postoperative pathological lymph node status (**Table 1**). Moreover, we also analyzed all breast cancer patients with clear information of lymph node status in TCGA cohort (characteristics were presented in **Table S2**). It was found that the expression of Linc00665 was positively correlated with lymph node metastasis. In the high-Linc00665 group, the ratio of lymph node metastasis is 55.3% (278/503), while the corresponding ratio was 48.3% (243/503) in the low-Linc00665 group (P = 0.027, **Figure S1**).

**Table 1:** Correlation between Linc00665 expression and clinicopathological features in breast cancer.

Variables		Low Linc00665 expression (n = 51)	High Linc00665 expression (n = 51)	P value
**Age**		50.98 (9.94)*	53.04 (10.34)*	0.308
**HR status**	Negative	16 (31.4%)	10 (19.6%)	0.256
	Positive	35 (68.6%)	41 (80.4%)	
**HER2 status**	Negative	24 (47.1%)	33 (64.7%)	0.111
	Positive	27 (52.9%)	18 (35.3%)	
**Ki67 level**	≤40%	24 (47.1%)	19 (37.3%)	0.423
	>40%	27 (52.9%)	32 (62.7%)	
**Tumor size**	≤5cm	12 (23.5%)	7 (13.7%)	0.309
	>5cm	39 (76.5%)	44 (86.3%)	
**Clinical lymph node status before chemotherapy**	Negative	4 (7.8%)	6 (11.8%)	0.739
	Positive	47 (92.2%)	45 (88.2%)	
**Menopausal status**	Premenopausal	25 (49.0%)	21 (41.2%)	0.551
	Postmenopausal	26 (51.0%)	30 (58.8%)	
**Pathological lymph node status**	Negative	35 (68.6%)	20 (39.2%)	0.003
	Positive	16 (31.4%)	31(60.8%)	
**NAC response**	Non-pCR	27 (52.9%)	40 (78.4%)	0.012
	pCR	24 (47.1%)	11 (21.6%)	

*mean (SD).

HR, hormone receptor; HER2, human epidermal growth factor receptor; NAC, neoadjuvant chemotherapy; pCR, pathological complete response.

### Linc00665 Is a Predictive Marker for Evaluating pCR After NAC in All Breast Cancer Patients

**Figure 1:**
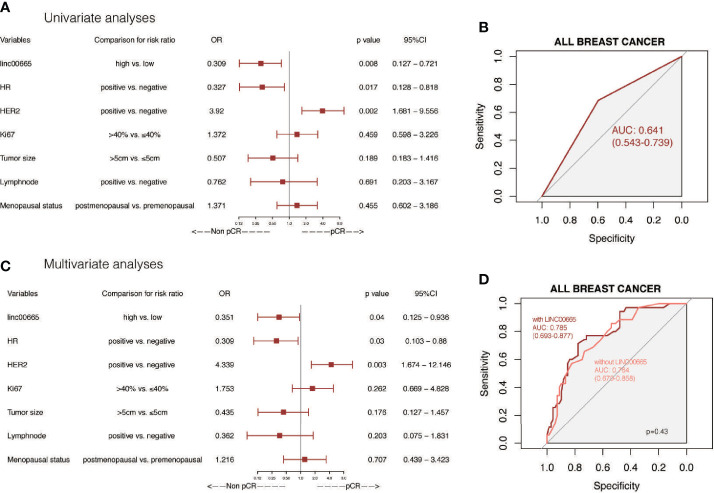
The predictive value of Linc00665 expression in NAC pCR in all patients included **(A)** Univariate logistic regression analysis of potential predictive markers in the whole cohort; **(B)** Performance of Linc00665 expression in predicting pCR evaluated by ROC in the whole cohort; **(C)** Multivariate logistic regression analysis of potential predictive markers in the whole cohort; **(D)** ROC of multivariate logistics regression including clinicopathological factors with or without Linc00665 expression in the whole cohort.

The pCR rate was 47.1% in the low-Linc00665 group and 21.6% in the high-Linc00665 group (P = 0.012, **Table 1**). The univariate analysis indicated that Linc00665 expression (OR = 0.309, P = 0.008, 95% CI: 0.127–0.721) was a predictor for pCR in breast cancer patients after NAC (**Figure 1A**), with AUC 0.641, as shown in **Figure 1B**. In addition, HR positive (OR = 0.327, P = 0.017, 95% CI: 0.128–0.818) and HER2 positive (OR = 3.920, P = 0.002, 95% CI: 1.681–9.556) were also predictive indicators for pCR (**Figure 1A**). The multivariate analysis of Linc00665 and pCR adjusted by HR status, HER2 status, Ki67 level, tumor size, lymph node status and menopausal status suggested that Linc00665 expression was an independent predictor for pCR (OR = 0.351, P = 0.040, 95% CI: 0.125–0.936). Besides, HR expression was negatively correlated with pCR (OR = 0.309, P = 0.030, 95% CI: 0.103–0.880), while the HER2 expression was positively correlated with pCR (OR = 4.339, P = 0.030, 95% CI: 1.674–12.146), as shown in **Figure 1C**. With the relatively higher AUC 0.785 (95% CI: 0.693–0.877) in ROC curve, the multivariate model that combined Linc00665 and the clinicopathological factors exhibited a better performance, when compared to the model with only the clinicopathological factors and an AUC of 0.764 (95% CI: = 0.670–0.858), as shown in **Figure 1D**.

### Subgroup Analysis and Interaction Analysis Between Linc00665 and Clinicopathological Factors on pCR After NAC

**Figure 2:**
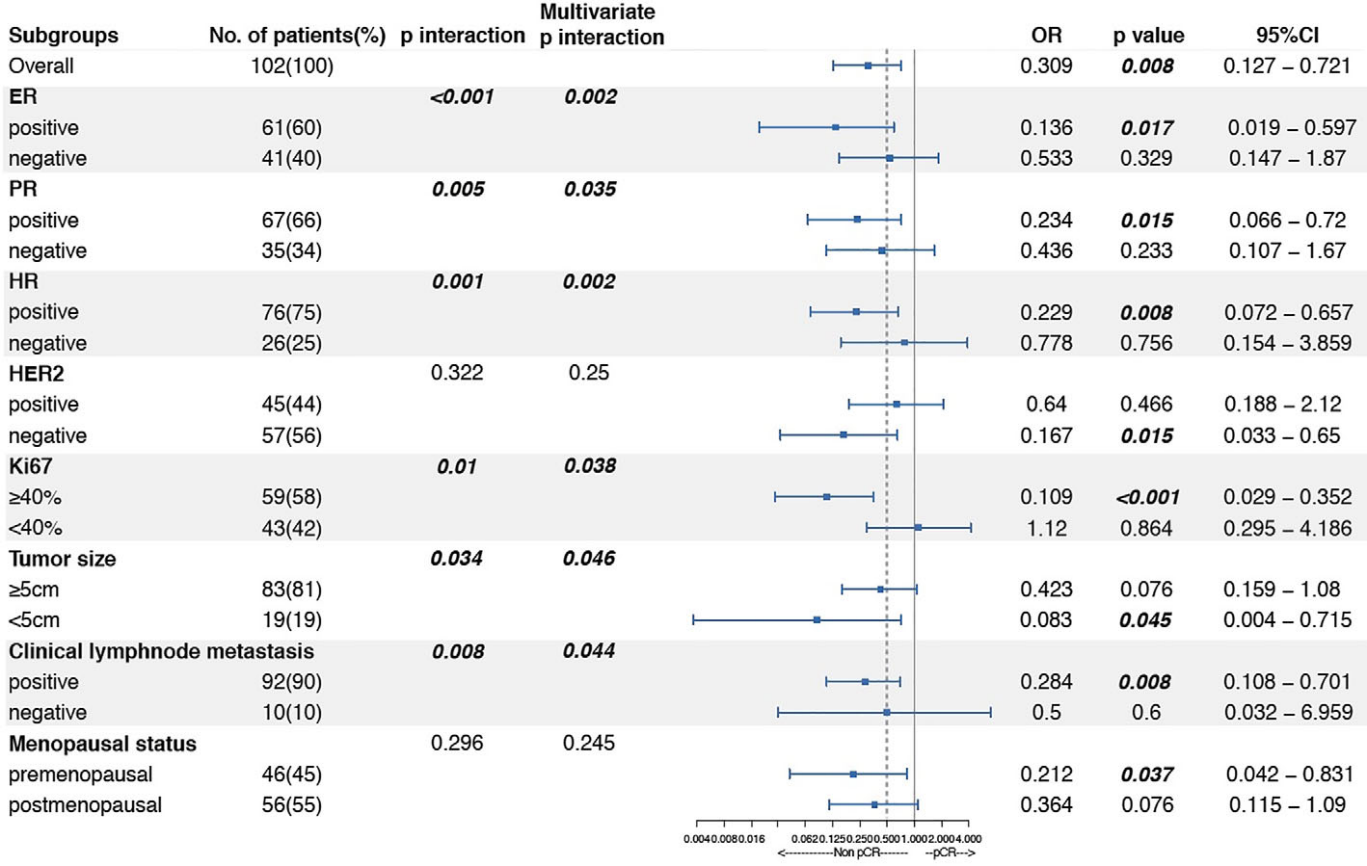
Subgroup analysis and interaction analysis between Linc00665 and clinicopathological factors regarding pCR after NAC.

**Figure 3:**
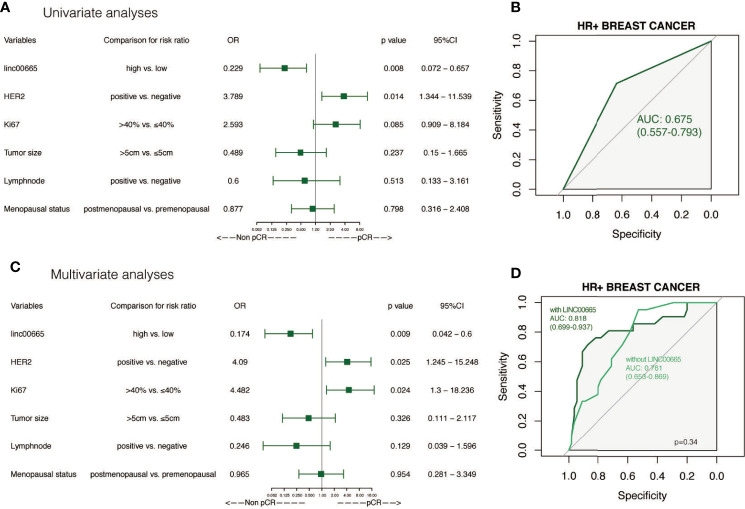
The predictive value of Linc00665 expression in pCR in HR-positive population **(A)** Univariate logistic regression analysis of potential predictive markers in HR-positive patients; **(B)** Performance of Linc00665 expression in predicting pCR evaluated by ROC in HR-positive patients; **(C)** Multivariate logistics regression analysis of potential predictive markers in HR-positive patients; **(D)** ROC of multivariate logistic regression including clinicopathological factors with or without Linc00665 expression in HR-positive patients.

**Figure 4:**
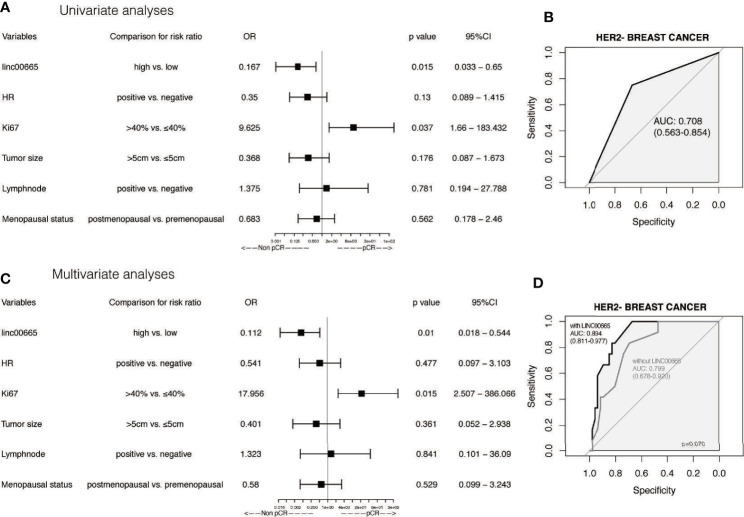
The predictive value of Linc00665 expression in pCR in HER2-negative patients **(A)** Univariate logistic regression analysis of potential predictive markers HER2-negative patients; **(B)** Performance of Linc00665 expression in predicting pCR evaluated by ROC in HER2-negative patients; **(C)** Multivariate logistic regression analysis of potential predictive markers in HER2-negative patients; **(D)** ROC of multivariate logistic regression including clinicopathological factors with or without Linc00665 expression in HER2-negative patients.

Linc00665 and ER exhibited a strong interaction in the univariate and multivariate analysis (univariate P < 0.0012, multivariate P = 0.002) regarding pCR after NAC. Similarly, Linc00665 and PR also exhibited an interaction in the univariate and multivariate analysis (univariate P = 0.005, multivariate P = 0.035). The interaction analysis of Linc00665 and HR was also significant (univariate P = 0.001, multivariate P = 0.002), as shown in **Figure 2**. For HR positive patients, the predictive value of Linc00665 for pCR was statistically significant in the univariate analysis (OR = 0.229, P = 0.008, 95% CI: 0.072–0.657, **Figure 3A**) with AUC 0.675 (**Figure 3B**) and multivariate analysis (OR = 0.174, P = 0.009, 95% CI: 0.042–0.600, **Figure 3C**) with AUC 0.818 (**Figure 3D**), which implies that Linc00665 is an independent predictive factor for HR-positive patients, but not for HR-negative patients. In HER2-negative patients, the univariate analysis showed that Linc00665 was tightly associated with the pCR rate (OR = 0.167, P = 0.015, 95% CI: 0.033–0.650, **Figure 4A**) with AUC 0.708 (**Figure 4B**). Similarly, the multivariate analysis (OR = 0.112, P = 0.010, 95% CI: 0.018–0.544, **Figure 4C**) with AUC 0.894 (**Figure 4D**) indicated that Linc00665 can independently predict the response to NAC. However, we did not find the interaction between Linc00665 and HER2 status (univariate P = 0.322, multivariate P = 0.250, **Figure 2**).

**Figure 5:**
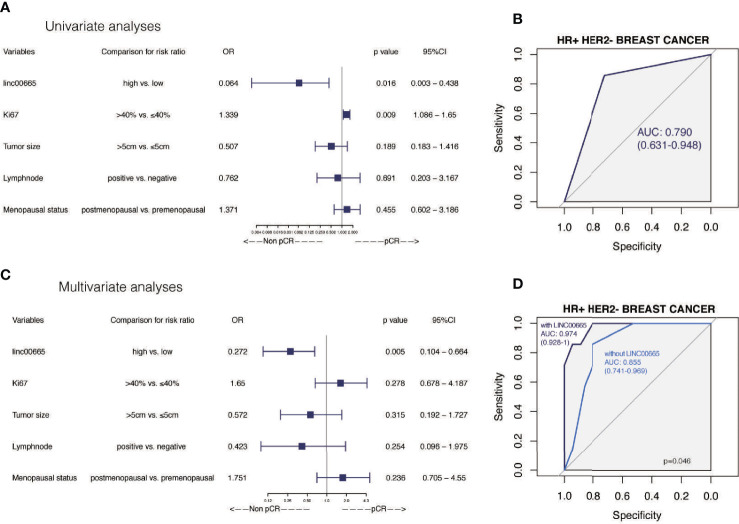
The predictive value of Linc00665 expression in pCR in HR-positive/HER2-negative patients **(A)** Univariate logistic regression analysis of potential predictive markers in HR-positive/HER2-negative patients; **(B)** Performance of Linc00665 expression in predicting pCR evaluated by ROC in HR-positive/HER2-negative patients; **(C)** Multivariate logistic regression analysis of potential predictive markers in HR-positive/HER2-negative patients; **(D)** ROC of multivariate logistic regression including clinicopathological factors with or without Linc00665 expression in HR-positive/HER2-negative patients.

Given the predictive role of Linc00665 in HR-positive and HER2-negative patients, respectively, we further analyzed Linc00665 expression for predicting the pCR rate in HR-positive/HER2-negative subtype. Interestingly, the predictive value of Linc00665 was observed with statistical significance in both univariate (OR = 0.064, P = 0.016, 95% CI: 0.003–0.438, **Figure 5A**) with AUC 0.790 (**Figure 5B**) and multivariate analysis (OR = 0.272, P = 0.005, 95% CI: 0.104–0.664, **Figure 5C**) with AUC 0.974 (**Figure 5D**).

However, in HR-positive, HER2-negative and HR-positive/HER2-negative subgroups, when the predictive model was established only by clinicopathological characteristics, the AUC was 0.761 (95% CI: 0.653–0.869, **Figure 3D**), 0.799 (95% CI: 0.678–0.920, **Figure 4D**) and 0.855 (95% CI: 0.741–0.969, **Figure 5D**), respectively.

In addition, Linc00665 also interacted with Ki67 (univariate P = 0.010, multivariate P = 0.038), tumor size (univariate P = 0.034, multivariate P = 0.046) and lymph node metastasis (univariate P = 0.008, multivariate P = 0.044) in terms of pCR. The correlation between Linc00665 and pCR was statistically significant in the patients with Ki67≥40% (OR = 0.109, P < 0.001, 95% CI: 0.029–0.352), tumor size <5cm (OR = 0.083, P = 0.045, 95% CI: 0.004–0.715) and clinical lymph node metastasis (OR = 0.284, P = 0.008, 95% CI: 0.108–0.701). Furthermore, Linc0065 was significantly correlated with pCR in the premenopausal subgroup (OR = 0.212, P = 0.037, 95% CI: =0.042–0.831), but not in the postmenopausal subgroup. However, no interaction was observed between Linc0065 and the menopausal status (P > 0.05), as shown in **Figure 2**.

### Gene Enrichment Pathways That Linc00665 Participates in

**Figure 6:**
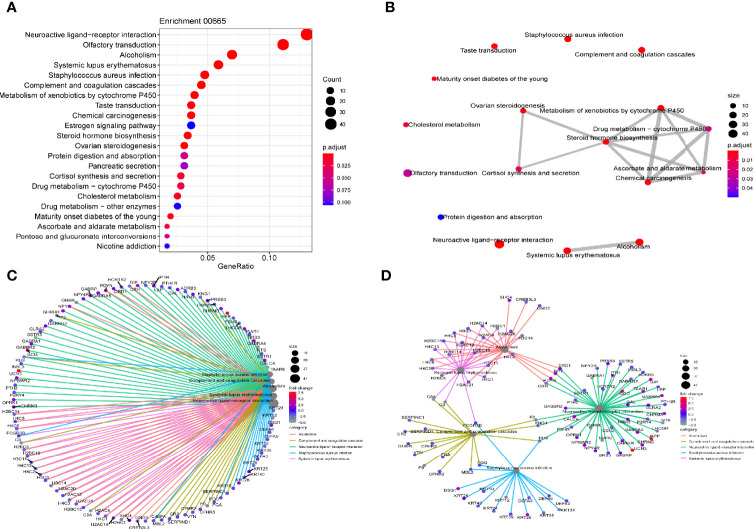
KEGG pathway analysis **(A)** Dot plot reflects most enriched pathways; **(B)** Map plot reflects relationship between enriched pathways; **(C, D)** Net plot indicates DEG (differential expressed gene) distribution in different enriched pathways.

**Figure 7:**
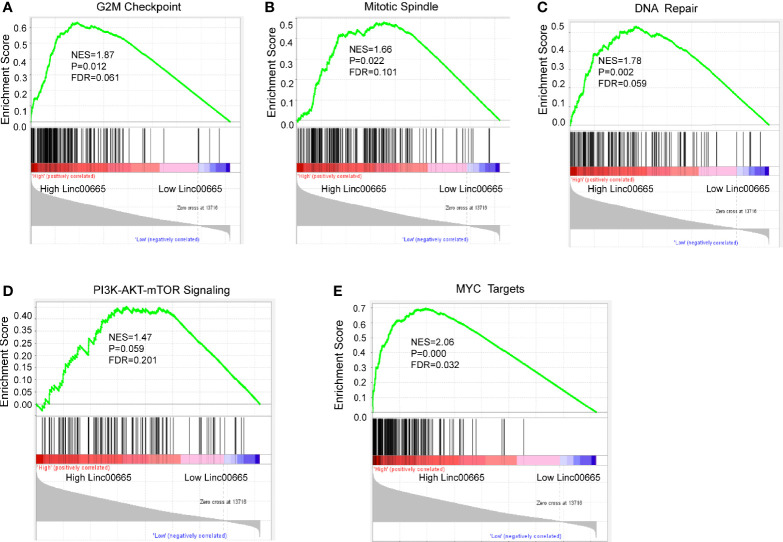
Enrichment plots from GSEA analysis. The Cancer Genome Atlas (TCGA) RNA-seq data were segmented into two groups on the basis of Linc00665 expression. GSEA plot was based on the gene expression profiles of Linc00665-high group compared with Linc00665-low group. NES, normalized enrichment score. False discovery rate (FDR) was set at 0.25. GSEA analysis suggested that different signaling pathways were enriched in Linc00665 high expression phenotype. **(A)** G2M checkpoint; **(B)** Mitotic Spindle; **(C)** DNA repair; **(D)** PI3K/AKT.MTOR signaling pathway; **(E)** MYC targets.

KEGG and GSEA signaling pathway analyses were conducted to determine the underlying molecular and biological processes Linc00665 potentially take part in. We identified a total of 4,459 differentially expressed genes (|logFC|>2, FDR<0.25) in TCGA breast cancer cohort by differential expression gene analysis. The result for the KEGG enrichment pathway analysis demonstrated that Linc00665 regulating pathways mainly focused on steroid hormone biosynthesis, ovarian steroidogenesis and drug metabolism-related pathway (**Figure 6**). To further understand the biological functions of Linc00665 in breast cancer, we performed GSEA enrichment analysis on high and low expression group of Linc00665 in TCGA dataset. The consequence revealed significant differences (FDR<0.25). Furthermore, we screened for the most notably enriched signaling pathways based on their Normal Enrichment Score (NES). A number of tumor-related pathways including the G2/M checkpoint (**Figure 7A**), Mitotic spindle (**Figure 7B**), DNA repair (**Figure 7C**), PI3K-AKT-mTOR signaling pathway (**Figure 7D**) and MYC targets (**Figure 7E**) were differentially concentrated in the Linc00665 high expression phenotype.

## Discussion

Linc00665 is a novel and poorly investigated lncRNA (13). Our study clarified the association between Linc00665 expression and response to NAC in breast cancer patients. To the best of our knowledge, this is the first study to report Linc00665 as a novel biomarker to predict chemosensitivity for breast cancer in the neoadjuvant setting.

Our study revealed for the first time that patients with high Linc00665 expression were less likely to achieve pCR after NAC. This means that Linc00665 overexpression might lead to insensitivity or resistance to chemotherapy. However, what is the possible underlying mechanism behind it in breast cancer patients? Yu et al. reported that overexpression of Linc00665 can promote breast cancer cells to acquire EMT phenotype, and a number of researches have been conducted on the relationship between EMT and drug resistance of cancer cells. For example, doxorubicin resistant MCF-7 cell lines and vincristine resistant ZR-75-B cell lines acquired EMT phenotypes (12). Subsequent studies have confirmed that the acquisition of chemoresistance is often accompanied by the acquisition of EMT phenotype in a variety of cancers, including breast cancer and pancreatic cancer (17). *In vivo* experiments in mice have also confirmed that EMT plays an important role in drug resistance in tumor and metastasis after chemotherapy (18). Our previous study also proved that patients with high expression of ZEB1, which is an important molecule in EMT, were less likely to achieve pCR after NAC (19). Therefore, Linc00665 may mediate chemoresistance by promoting EMT.

Besides EMT mechanism, it has been reported that Linc00665 overexpression was involved in gefitinib resistance in NSCLC by activating PI3K/Akt pathway (14). Inhibition of PI3K/Akt/mTOR overcomes cisplatin resistance in the triple negative breast cancer cell line HCC38 (20). Feng et al. suggested that inhibition of PI3K/Akt/mTOR pathway in MCF-7 cells can enhance the chemosensitivity of breast cancer cells to cisplatin by enhancing autophagy and apoptosis (21). Chi et al. demonstrated that CapG has been shown to increase PIK3R1 expression and activate the PI3K/Akt pathway, mediating resistance to paclitaxel in breast cancer patients (22). Through GSEA analysis, we found that Linc00665 overexpression can activate PI3K/Akt pathway. He et al. demonstrated that Linc00665 may promote the progression of hepatic cellular cancer by activating NF-κB signaling pathway, and the activation of NF-κB pathway may reduce the sensitivity of MDA-MB-231 cells to doxorubicin (23). KEGG analysis showed that Linc00665 participates in drug metabolism cytochrome P450 pathway. Serdina et al. reported that CYP2C9*2 gene polymorphism of cytochrome P450 family members was positively correlated with NAC drug resistance after receiving CMF (Cyclophosphamide, Methotrexate and Fluorouracil) or CMXeloda (Cyclophosphamide, Methotrexate and Xeloda), FAC (Fluorouracil, Adriamycin and Cyclophosphamide) and CAF (Cyclophosphamide, Adriamycin and Fluorouracil) or CAXeloda (Cyclophosphamide, Adriamycin and Xeloda) NAC reagents (24). What’s more, it has been suggested that Y-box binding protein-1 (YB-1) may be involved in the drug resistance of cisplatin (25), paclitaxel (26), and Adriamycin (27), indicating that Linc00665 can promote the growth of lung adenocarcinoma by interacting with YB-1 (28). It is also well-known that cisplatin induces tumor cell death by inducing DNA damage, and paclitaxel leads to cell proliferation arrest and cell death by inhibiting microscopic dynamics and activating mitotic checkpoint (29). Interestingly, GSEA analysis showed that Linc00665 was positively correlated with G2/M, DNA repair and mitotic spindle. It is reasonable to speculate that Linc00665 may enhance the ability of breast cancer cells to repair DNA damage and promote the spindle assembly process by stimulating G2/M, thus resulting in poor response to NAC. All studies above directly or indirectly support the hypothesis that Linc00665 overexpression may mediate the chemo-insensitivity of breast cancer NAC through multiple pathways. This may be induced through triggering PI3K/AKT, NF-κB and YB-1 signaling pathways or through affecting drug metabolism, G2/M and DNA repair to induce chemotherapy resistance. All these mentioned potential molecular mechanisms deserve further exploration.

Our study found that Linc00665 expression in the HR-positive/HER2-negative subgroup was of more predictive value for pCR after NAC. We also observed that Linc00665 interacts with the HR status through the interaction analysis, which indicates that different HR status may affect Linc00665 function. Zhou et al. reported that Linc00665 can encode a micropeptide cip2a-bp and inhibit PI3K/Akt/NF-κB pathway in triple negative breast cancer, thus inhibiting the invasion and metastasis of triple negative breast cancer (30). Yu et al. reported higher expression of Linc00665 in triple negative breast cancer cells when compared with ER+ breast cancer cells (12). These two basic studies suggested that different HR status might not only affect the expression level of Linc00665 but also function by different mechanism. In addition, through KEGG analysis, we also found that Linc00665 was involved in the regulation of ER pathway. All these might at least partly explain the complex interaction between Linc00665 and the ER pathway, making Linc00665 a novel predictive biomarker for breast cancer especially in HR-positive patients.

We found Linc00665 expression level was positively correlated to postoperative pathological lymph node status. This implied that Linc00665 may possess the ability to promote breast cancer metastasis. Previous studies have shown that overexpression of Linc00665 can enhance the invasion and metastasis of various tumors, which supports our speculation. Yu et al. reported that Linc00665 deletion can inhibit the migration and invasion of triple negative breast cancer cell lines MDA-MB-231 and BT549 (12, 31). Gao et al. elucidated that Linc00665 knockdown can inhibit the migration and invasion of gastric cancer cell lines AGS and BGC‐823 (9). Wang et al. also reported that Linc00665 silencing can significantly reduce the invasive ability of gastric cancer cell lines AGS and MGC-803 (32). Qiang et al. confirmed that knockdown of Linc00665 can inhibit the invasion ability of lung adenocarcinoma cell lines A549 and H1299 through the *in vitro* experiments. The results of subcutaneous tumor model in nude mice showed that lung metastases were significantly reduced in Linc00665 knockdown group (10). Zhang et al. have shown that Linc00665 deficiency can reduce the invasiveness of NSCLC cell lines A549 and H1299 (33). Overexpression of Linc00665 in T47D cell line can make it obtain EMT-like phenotype (12). Consistently, TCGA analysis shows that patients with high Linc00665 expression have an increased rate of lymph node metastasis, suggesting that tumor with high Linc00665 level may be more malignant. However, the correlation failed to be observed between Linc00665 expression and clinical lymph node status in our neoadjuvant cohort. Therefore, expanding the sample size to further clarify this issue may be extremely helpful, especially in patients without NAC.

There were several limitations of this study. First, due to the limited number of patients participating in this trial, we have not been able to analyze the correlation between Linc00665 expression and NAC sensitivity in triple negative breast cancer patients. We will further expand the sample size to analyze the predictive value of Linc00665 expression for NAC response in triple negative breast cancer patients. Second, since the follow-up time is relatively short, the survival analysis is not mature. However, our study is a retrospective study based on a prospective cohort study. The predictive value of Linc00665 expression for NAC is still of great clinical significance. Expanding the sample size and further follow up to clarify the predictive and prognostic value of Linc00665 in breast cancer are required in the future study.

## Conclusion

In summary, our study revealed that Linc00665 expression is a negative predictor for pCR in the neoadjuvant setting, especially for HR-positive/HER2-negative patients, which can serve as a novel predictive biomarker for the chemosensitivity. However, specific molecular mechanisms behind the findings between Linc00665 and NAC sensitivity need further study.

## Data Availability Statement

The datasets presented in this study can be found in online repositories. The names of the repository/repositories and accession number(s) can be found in the article/**Supplementary Material**.

## Ethics Statement

The studies involving human participants were reviewed and approved by institutional review board of Renji Hospital, School of Medicine, Shanghai Jiao Tong University. The patients/participants provided their written informed consent to participate in this study.

## Author Contributions

JL and WY designed the study. HD performed qRT-PCR. HD and XS drafted the manuscript. XS and YX conducted the statistical analysis. YL, ZL, FY, and RS collected sample tissues and clinical information. RS, SZ, and JP extracted RNA. JL revised the manuscript. All authors contributed to the article and approved the submitted version.

## Funding

This work was supported by the National Natural Science Foundation of China [Grant Numbers 81172505, 81302302, 82002777 and 81972854], Doctoral Programs Foundation of the Ministry of Education of China [Grant Number 20120071120105], Shanghai Natural Science Foundation [Grant Numbers 13ZR1452800 and 19ZR1431100], Shanghai Municipal Commission of Health and Family Planning [Grant Numbers 20144Y0218 and 201640006], Clinical Research Plan of Shanghai Hospital Development Center [Grant Numbers 16CR3065B and 12016231], Shanghai “Rising Stars of Medical Talent” Youth Development Program for Outstanding Youth Medical Talents [Grant Number 2018-16], Shanghai Collaborative Innovation Center for Translational Medicine [Grant Number TM201908], Multidisciplinary Cross Research Foundation of Shanghai Jiao Tong University [Grant Numbers YG2017QN49, ZH2018QNA42 and YG2019QNA26], Nurturing Fund of Renji Hospital [Grant Number PYMDT-002, PY2018-III-15 and PYIII20-09], Science and Technology Commission of Shanghai Municipality [Grant Number 15JC1402700], and Shanghai Municipal Key Clinical Specialty. the Clinical Research Plan of SHDC (Grant Number 16CR3065B, 1201623).

## Conflict of Interest

The authors declare that the research was conducted in the absence of any commercial or financial relationships that could be construed as a potential conflict of interest.

## Supplementary Material

Supplementary Figure 1 Association between Linc00665 expression and lymph node status in The Cancer Genome Atlas (TCGA) cohort.
