# Supplementary figures and images for: Linc00665 Can Predict the Response to Cisplatin-Paclitaxel Neoadjuvant Chemotherapy for Breast Cancer Patients
Source: Front Oncol. 2021 Mar 2;11:604319. doi: 10.3389/fonc.2021.604319 (PMC7961084; doi:10.3389/fonc.2021.604319)

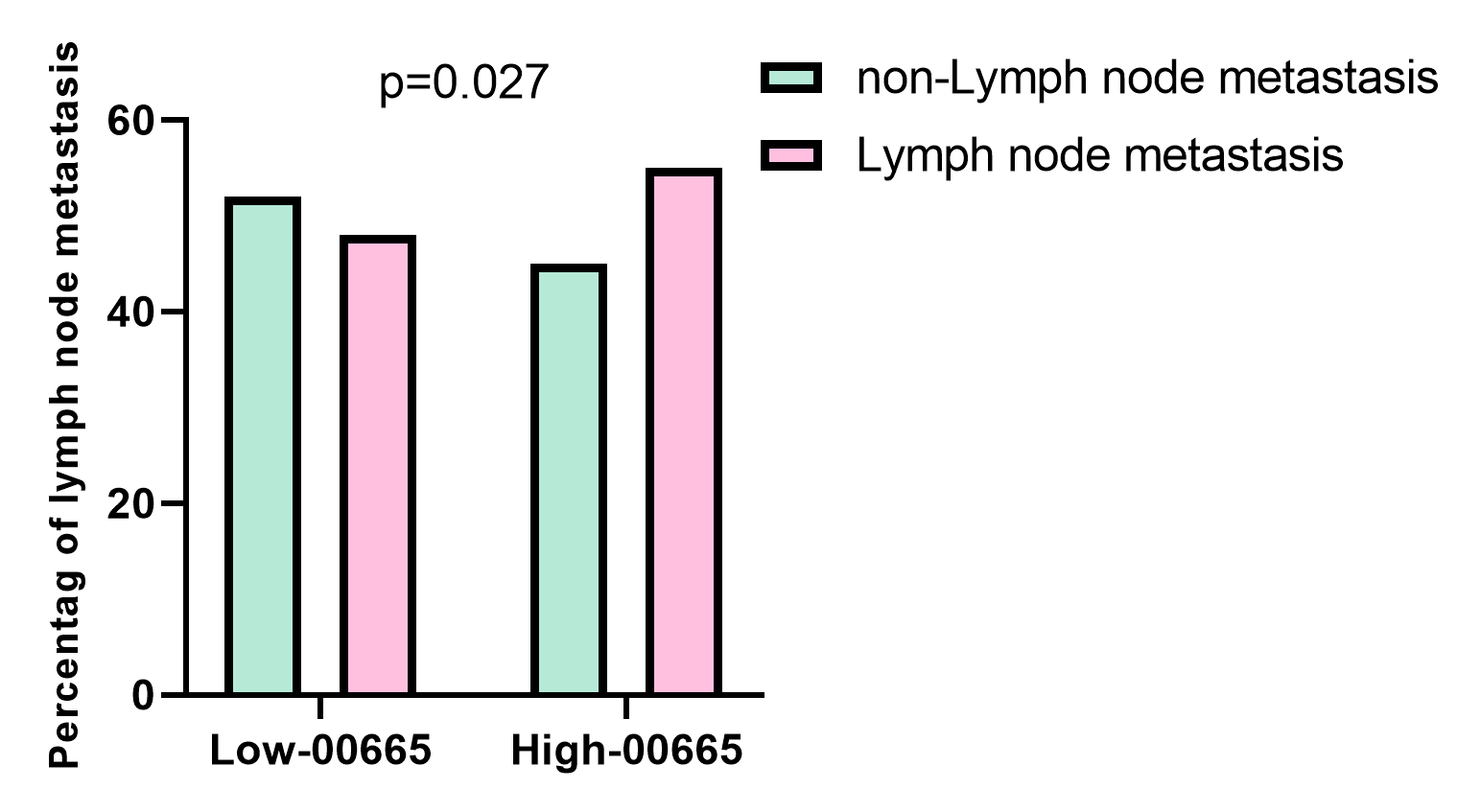

Supplement: Supplementary Figure 1 — Association between Linc00665 expression and lymph node status in The Cancer Genome Atlas (TCGA) cohort. [file Image_1.tif]
