# Supplementary material for: Linc00665 Can Predict the Response to Cisplatin-Paclitaxel Neoadjuvant Chemotherapy for Breast Cancer Patients
Source: Front Oncol. 2021 Mar 2;11:604319. doi: 10.3389/fonc.2021.604319 (PMC7961084; doi:10.3389/fonc.2021.604319)
Supplement: Supplementary file 2 [file Table_1.docx]

Supplementary Table 1 Primers employed for RT- PCR in this study

| Primer name | Primer sequence (5’-3’) |
| --- | --- |
| β-actin-F | CATGTACGTTGCTATCCAGGC-3' |
| β-actin-R | CTCCTTAATGTCACGCACGAT |
| Linc00665-F | AACTACATCAGCGCCAGTGC |
| Linc00665-R | CGTGGAGTCCTGGCCTTTTG |
