# Supplementary material for: Linc00665 Can Predict the Response to Cisplatin-Paclitaxel Neoadjuvant Chemotherapy for Breast Cancer Patients
Source: Front Oncol. 2021 Mar 2;11:604319. doi: 10.3389/fonc.2021.604319 (PMC7961084; doi:10.3389/fonc.2021.604319)
Supplement: Supplementary file 3 [file Table_2.docx]

**Table S2** Association between Linc00665 expression and clinicopathological characteristics in TCGA breast cancer patients

| Variables | | Low Linc00665 expression (n=503) | High Linc00665 expression (n=503) | P value |
| --- | --- | --- | --- | --- |
| Age |  | 59.78 (±13.31) | 58.21 (±12.89) | 0.059 |
| ER status | Negative | 90 (18.9%) | 144(30.8%) | <0.001 |
|  | Positive | 387 (81.1%) | 323(69.2%) |  |
|  | Not applicable | 26 | 36 |  |
| PR status | Negative | 132 (27.2%) | 186 (38.9%) | <0.001 |
|  | Positive | 354 (72.8%) | 292 (61.1%) |  |
|  | Not applicable | 17 | 25 |  |
| HR status | Negative | 68 (14.3%) | 122 (26.1%) | <0.001 |
|  | Positive | 409 (85.7%) | 345 (73.9%) |  |
|  | Not applicable | 26 | 36 |  |
| HER2 status | Negative | 238 (70.0%) | 271(80.2%) | 0.003 |
|  | Positive | 102 (30.0%) | 67 (19.8%) |  |
|  | Not applicable | 163 | 165 |  |
| Tumor size | ≤5cm | 421 (83.7%) | 429 (85.3%) | 0.542 |
|  | >5cm | 82 (16.3%) | 74 (14.7%) |  |
| Lymph node status | Negative | 260 (51.7%) | 225 (44.7%) | 0.032 |
|  | Positive | 243 (48.3%) | 278 (55.3%) |  |

Abbreviations: ER, estrogen receptor; PR, progesterone receptor; HR, hormone receptor; HER2, human epidermal growth factor receptor 2.
